# Improving Diagnostic Accuracy in Acute Pulmonary Embolism: Insights from Spectral Dual-energy CT

**DOI:** 10.2174/0115734056323974241202175740

**Published:** 2025-01-02

**Authors:** Mei-Ling Shen, Han-Wen Zhang, Li-Hong Liu, Wei-Ming Liu, Hua Zhong, Biao Huang, Yu-li Wang, Fan Lin

**Affiliations:** 1 Department of Radiology, Shantou University Medical College, No. 22, Xinling Road, Shantou City, China; 2 Department of Radiology, Kuichong People's Hospital, Dapeng New District, Shenzhen 518119, China; 3 Department of Radiology, The First Affiliated Hospital of Shenzhen University, Health Science Center, Shenzhen Second People’s Hospital,3002 SunGangXi Road, Shenzhen, China; 4 Department of Radiology, Guangdong Provincial People’s Hospital (Guangdong Academy of Medical Sciences), Southern Medical University, 106 Zhongshan 2nd Road, Guangzhou, Guangdong, China.

**Keywords:** Spectral dual-energy detector computed tomography (SDCT), Acute pulmonary embolism, CT angiography (CTA), Clinical imaging diagnosis, Pulmonary embolism, Virtual monoenergetic images (VMIs)

## Abstract

**Purpose:**

This study aims to evaluate the clinical efficacy of spectral dual-energy detector computed tomography (SDCT) and its associated parameters in diagnosing acute pulmonary embolism (APE).

**Methods:**

Retrospective analysis of imaging data from 86 APE-diagnosed patients using SDCT was conducted. Virtual monoenergetic images (VMIs) at 40, 70, and 100 KeV, Iodine concentration (IC) maps, Electron Cloud Density Map (ECDM), Effective atomic number (Z-eff) maps, and Hounsfield unit attenuation plots (VMI slope) were reconstructed from pulmonary artery phase CT images. The subtraction (SUB) and ratios of VMIs were calculated, and two experienced radiologists evaluated the patients. The Mann-Whitney U test assessed the parameter ability to differentiate between normal and obstructed lung fields and detect emboli in the pulmonary artery. Receiver Operating Characteristic Curves (ROC) were generated for performance evaluation.

**Results:**

Significant differences (*p*<0.001) in 40KeV, Ratio, SUB, and Z-eff were found between normal and embolized lung fields. Logistic regression demonstrated good detection performance for Z-eff (AUC=0.986), SUB (AUC=0.975), and IC (AUC=0.974). Parameters such as 40KeV (AUC=0.990), 70KeV (AUC=0.980), 100KeV (AUC=0.962), SUB (AUC=0.990), Z-eff (AUC=0.999), and IC (AUC=1.000) exhibited good detection capabilities for identifying emboli in the pulmonary artery.

**Conclusion:**

SDCT facilitates the identification of diseased lung fields and localization of emboli in the pulmonary artery, thereby improving diagnostic efficiency in APE.

## 1 INTRODUCTION

Acute pulmonary embolism (APE) stands as the third most lethal cardiovascular affliction [1, 2], striking with an incidence rate ranging from 29 to 115 cases per 100,000 individuals [3]. Simultaneously, APE bears a high mortality rate, claiming over 100,000 lives annually in the United States alone [4]. However, with the relentless enhancement of imaging detection technologies and increased clinical focus on this malady, the diagnostic capabilities for detecting APE have seen steady improvement, consequently contributing to a gradual decline in mortality rates associated with APE[.3]

APE can arise from various clinical factors, including severe trauma, surgical procedures, immune-related conditions, diabetes, hypertension, and prolonged immobilization [5, 6]. The European Society of Cardiology (ESC) guidelines emphasize the importance of biomarkers in diagnosing, assessing risk, and predicting prognosis in APE.3 D-dimer (D-2) levels typically surge notably during acute thrombosis, serving as an indicator of APE when coupled with relevant patient symptoms [7, 8]. However, owing to the diverse sources of emboli, their compositions vary significantly, leading to differences in embolus density and lung imaging presentations on emergency scans. Enhancing the diagnostic accuracy of APE through imaging holds the potential to enhance clinical practice and facilitate improved treatments, such as arterial thrombolysis.

Pulmonary artery computed tomography angiography (CTA) is commonly employed in clinical settings for imaging detection to pinpoint the location and assess the severity of APE [9, 10]. Nonetheless, clinical scenarios frequently arise where diagnostic indicators suggest APE, yet CTA results are negative [11]. The virtual monoenergetic images (VMIs) produced by spectral dual-energy detector computed tomography (SDCT) exhibit superior soft tissue detection capabilities [12]. Additionally, the iodine concentration (IC) map, effective atomic number (Z-eff) map, and Hounsfield unit attenuation plot (VMI slope) enhance the detection of diagnostic information related to APE [13, 14].

SDCT theoretically enables the identification of lesion sites through corresponding technology, subsequently allowing for focused examination of the pulmonary artery to detect embolic sites. This study aims to retrospectively analyze the SDCT data in patients with acute pulmonary embolism from our hospital, seeking to explore the diagnostic clinical value of this technology for APE.

## 2 MATERIALS AND METHODS

### 2.1 Patients

This retrospective study received approval from the local ethics committee, with patients exempted from signing informed consent. Data from 86 patients diagnosed with pulmonary embolism and scanned using SDCT (Philips, IQon, Netherlands) at our center between January 2021 and May 2023 were collected. Inclusion criteria comprised patients with clinical test indicators suggestive of pulmonary embolism and spectral CT-enhanced scans capable of identifying intrapulmonary embolism. Exclusion criteria encompassed (1) CT images of inadequate quality for evaluation (*e.g*., metal artefacts), (2) Lesions too small to affect region of interest (ROI) selection (*e.g*., emboli in terminal small pulmonary artery branches), (3) Patients undergoing post-pulmonary embolism review, (4) Patients lacking substantial clinical data, including SBI data and other original evaluation imaging data. Ultimately, 86 patients were included, comprising 53 males (61.6%) and 33 females (38.4%), aged between 28 and 87 years (mean age: 63 ± 49 years).

### 2.2 SDCT Scanning Protocol

All patients underwent scanning using the IQon SDCT machine. Prior to the CT examination, patients were positioned supine with their heads first. The scanning range extended from the lung apex to the lung base. Scanning parameters included a collimator width of 64×0.625 mm, tube voltage of 120 kVp, tube current controlled automatically using DoseRight Index 20 (average Mas103), X-ray tube rotation speed of 0.27 s/cycle, and a pitch of 1.258. Reconstruction comprised a layer thickness of 0.9 mm with a spacing of 0.45 mm. Enhanced scans utilized contrast agent intelligent tracking threshold trigger technology, with the trigger point set in the right atrium and a trigger threshold of 70 Hounsfield Units (HU). Upon reaching the designated threshold, scanning commenced with a delay of 6-8 seconds. A conventional two-phase scan was performed, consisting of a first pulmonary artery phase scan followed by a venous phase scan. Iopromide (Beilu Pharmaceutical, China) (iodine concentration 370 mg/ml) was used as the contrast agent, administered at a dose of 30-50 ml with an injection flow rate of 4 ml/s.

### 2.3 Image Analysis Methods

All enrollment data were transferred to the Philips SpDS image workstation (Spectral Diagnostic Suite 6.5) for analysis, where Virtual Monoenergetic Images (VMI) were reconstructed based on CT pulmonary artery phase images. VMIs were reconstructed at intervals of 30 KeV within the range of 40~100KeV to obtain 40, 70, and 100KeV VMIs. Additionally, Z-eff, IC maps, and ECDM were obtained through post-processing, resulting in a total of 6 sets of images. Two radiologists, each with 6 and 10 years of experience, independently evaluated patient images. Their assessments included identifying pulmonary artery emboli, normal blood vessels, embolized lung fields, and normal lung fields, with corresponding Regions of Interest (ROI) delineated. The ROI outlining method involved selecting the largest layer of the embolus to outline the ROI while avoiding vessel edges. For embolized lung fields, a circular ROI was drawn, excluding blood vessels and inflammatory sites. If necessary, ROIs from normal lung fields in the contralateral lung segment were copied and pasted. Parameter values were measured, including Ratio=40KeV/100KeV and Subtraction (SUB)=40KeV – 100KeV to evaluate VMI parameter changes. Interclass Correlation Coefficient (ICC) consistency testing was conducted on all measurement data.

### 2.4 Statistical Analysis

Data statistics were conducted using the Statistical Package for the Social Sciences (SPSS v. 27, Chicago, Illinois). As the imaging data of normal lung fields, embolized lung fields, normal blood vessels, and emboli did not adhere to normal distribution, the Mann-Whitney U test was employed, with significance set at *p*<0.05. Univariate and multivariate analyzes were performed using logistic regression on these parameters. Receiver Operating Characteristic (ROC) curves were generated to illustrate performance. All statistical graphs were generated using GraphPad Prism 9 (GraphPad Software, La Jolla, California) (Table **1**) (supplementary material).

## 3 RESULTS

**Fig. (1):**
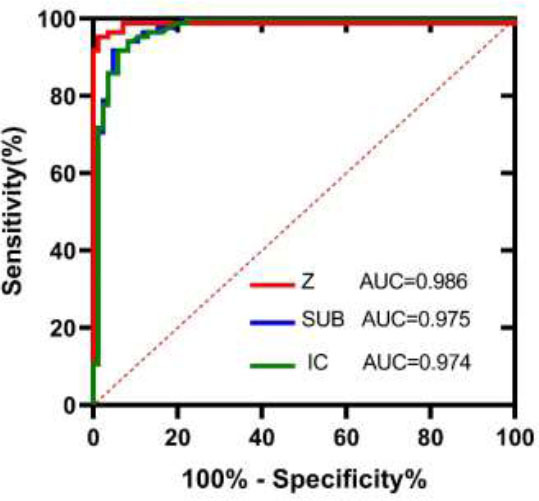
The ROC illustrates the ability of SDCT parameters to distinguish between normal lung fields and embolized lung fields.

Interobserver agreement between the two reviewers was excellent, with intraclass correlation coefficient (ICC) values exceeding 0.90 for all measurements. Mann-Whitney U test results between normal lung fields and embolized lung fields showed statistical significance for 40KeV, Ratio, SUB, Z-eff, and IC (*p*<0.001). Other clinical and imaging parameters did not exhibit statistical significance. Both single-factor and multi-factor analyses revealed that SUB, Z-eff, and IC were statistically significant (*p*<0.001). ROC analysis demonstrated that Z-eff (area under the curve, AUC=0.986), SUB (AUC=0.975), and IC (AUC=0.974) effectively distinguished normal lung fields from embolized lung fields (Fig. **1**).

**Fig. (2):**
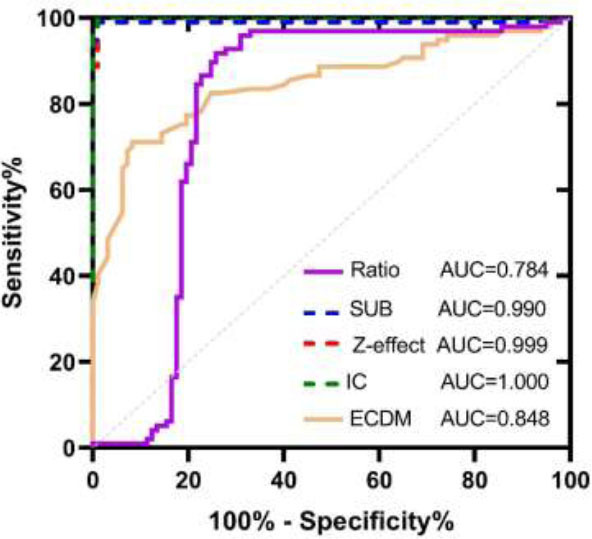
The ROC demonstrates the efficacy of SDCT parameters in identifying intravascular embolism within the embolized pulmonary artery.

Mann-Whitney U test results between normal pulmonary arteries and pulmonary artery thrombi indicated statistical significance for 40KeV, 70KeV, 100KeV, Ratio, SUB, Z-eff, IC, and ECDM (*p*<0.001). Single-factor and multi-factor analyses identified 40KeV, 70KeV, 100KeV, SUB, Z-effect, IC, and ECDM as statistically significant (*p*<0.001), while Ratio did not exhibit statistical significance. ROC analysis revealed good detection performance for all aforementioned parameters (AUC: 40KeV=0.990, 70KeV=0.980, 100KeV=0.962, SUB=0.990, Z-effect =0.999, IC=1.000, ECDM=0.848). These parameters are valuable for assisting in the identification of emboli in normal blood vessels (Fig. **2**) (Table **2**).

## 4 DISCUSSION

This study revealed that SDCT exhibits favorable performance both in detecting embolized lung fields and identifying intravascular emboli. Specifically, SUB, Z-eff, and IC can effectively distinguish normal lung fields from embolized lung fields. Moreover, 40KeV, 70KeV, 100KeV, SUB, Z-eff and ECDM demonstrate high efficacy in detecting emboli within the pulmonary artery. The findings suggest that dual-energy spectral detector CT holds promise in detecting pulmonary embolism lesions and assisting radiologists in emboli detection. Although different etiologies lead to minor variations in embolism, primarily in terms of composition and site of APE, there is an overlap among the causes, resulting in similar imaging appearances.

Due to variations among individuals and differences in the timing of embolization, simply measuring single-level CT values may not accurately reflect the corresponding lesion area. Analyzing the conversion of VMI can mitigate this difference to some extent and enable better differentiation between normal lung tissue and affected areas [13, 15]. During a conventional CT scan, the HU value typically corresponds to approximately 70KeV. Lowering energy levels enhances tissue contrast, and the disparity between low and high energy levels indirectly elucidates tissue structure within the region [16]. In our study, we observed statistical differences in 40KeV, SUB, and Ratio, indicating their potential to better capture this distinction. This finding contrasts with the spectral CT analysis of certain solid organs [17]. Although only SUB shows statistical significance after logistic regression, this is because both SUB and Ratio are interdependent factors, derived from CT values at 40KeV and 100KeV. This observation suggests that tissue discrepancies between normal lung tissue and diseased areas alter when APE occurs. Consequently, changes in Ratio and SUB further reflect intra-regional tissue disparities. The effective atomic number map provides additional diagnostic insights based on atomic composition, offering a visually impactful depiction of APE lesion areas. Meanwhile, the iodine density map facilitates quantitative analysis of iodine concentration (contrast agent concentration) within APE lesion regions.

**Fig. (3):**
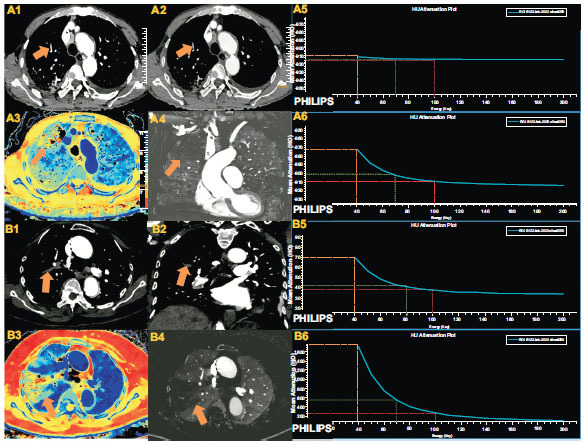
Imaging findings in two patients with acute pulmonary thromboembolism. **A**. Images from a 65-year-old male patient. A1-A2: Axial views at 40KeV show a clear embolus (arrow) in the anterior segment of the right upper lobe, not evident at 70KeV or on traditional pulmonary artery CTA. A3-A4: The atomic number map and iodine density map clearly depict the embolism in the anterior segment of the right upper lobe, presenting a fan-shaped lesion in the lung field (arrow). A5: Curve representing the embolized lung field. A6: Curve representing the normal lung field. **B**. Images from an 87-year-old female patient. B1-B2: Axial and coronal views at 40KeV display an embolus (arrow) in the posterior segment of the right upper lobe. B3-B4: The atomic number image and iodine density map reveal an embolism in the anterior segment of the right upper lobe, along with a fan-shaped lesion in the lung field. B5: Curve representing the embolus. B6: Curve representing a normal vessel.

In the context of detecting emboli within blood vessels, various parameters of SDCT exhibit excellent detection capabilities. Whether it's through alterations in VMI, disparities in IC, or the clear reflection of Z-eff, the distinction between blood and emboli within the pulmonary artery becomes notably apparent. Conventional CT scans may easily overlook diagnosing emboli that are smaller and possess a density akin to that of the pulmonary artery wall, thereby heightening the patient's risk. However, the detection of SDCT-related parameters effectively addresses this gap. 18 Our findings indicate that image detection efficiency at low energy levels surpasses that at high energy levels, aligning with the majority of SDCT studies. SDCT effectively delineates diseased areas initially, enabling subsequent emboli to search within the pulmonary arteries within these areas. This approach significantly alleviates radiologists' workload while enhancing APE emboli detection efficiency. In our study, we found that 96.51% of patients exhibited this fan-shaped change. The fan-shaped change appears as a fan-shaped shadow near the peripheral lung adjacent to the pleura, with its apex connected to the terminal part of the pulmonary artery, representing infarction of the lung caused by occlusion at the terminal part of the pulmonary artery. The mechanism of formation is that the embolism of the blood vessel supplying the lung segment leads to exudative consolidation in that segment. On imaging, the extent of consolidation is distributed along the lung segment supplied by the embolized vessel, forming a fan-shaped shadow with the apex pointing towards the hilum. Its clinical significance is that when there is a thrombus in the pulmonary artery, the fan-shaped change indicates that the lung segment has infarcted due to ischemia. Our study primarily focuses on quantitative parameter research, and we will further conduct subjective image scoring in the future (Fig. **3**).

Currently, dual-energy CT (DECT) is extensively employed in the diagnosis of APE. Hong *et al*. conducted a study on the utility of dual-energy CT in diagnosing pulmonary embolism. Their findings revealed that the sensitivity and specificity of dual-energy CT in diagnosing APE ranged from 60 to 90% and 88 to 99%, respectively [15]. In their study, the primary advantage of dual energy is its capability to display perfusion defects in lung parenchyma due to pulmonary artery occlusion and to directly observe filling defects in the pulmonary artery (PA). The iodine map efficiently demonstrates perfusion defects and allows for quantitative analysis of their volume. In addition, our study corroborates these findings, indicating that the IC map, VMI, and related parameters also facilitate quantitative analysis of APE. Langius-Wiffen *et al*. assessed iodine density using a deep learning (DL) algorithm. In a single-center application, the diagnostic efficiency for APE reached 96% (95% CI 0.92-0.98), and it reached 89% (95% CI 0.81-0.94) in the external validation set. These findings demonstrate the potential of the dual-energy CT deep learning algorithm in detecting pulmonary embolism, aligning closely with the results obtained in our study [18, 19]. However, our study did not involve engineering-related algorithms and is more closely related to the actual clinical applications, providing a detailed diagnostic process that aids in clinical diagnosis. In addition, Computed Tomography Pulmonary Angiography (CTPA) is the gold standard technique for diagnosing APE. Studies have demonstrated that CTPA exhibits a sensitivity of 83%, a specificity of 96%, and a positive predictive value of 86% for diagnosing PE [20]. Compared to DECT, CTPA involves a higher radiation dose and exhibits a lower detection rate for subsegmental pulmonary embolism, often failing to detect small emboli. In contrast, SDCT can enhance the examination of the current area through prompts from the atomic number map, thereby improving the detection of emboli.

Another commonly utilized diagnostic technique for pulmonary embolism is ventilation/perfusion single-photon emission computed tomography (V/Q SPECT). This method overcomes the challenge of superimposing areas of the lung with normal perfusion onto regions of perfusion defect. Numerous studies have showcased enhanced sensitivity and specificity of V/Q SPECT when compared to planar V/Q scintigraphy [21]. However, V/Q SPECT may not always accurately differentiate APE from other lung diseases, particularly in low-probability cases, and patients may be subjected to higher radiation doses during the examination. Additionally, V/Q SPECT offers lower spatial resolution, resulting in a lack of detailed anatomical information that may impede the assessment of pulmonary embolism location and size [22]. Indeed, DECT offers a higher contrast-to-noise ratio (CNR), allowing for the simultaneous acquisition of anatomical and functional information in a single scan. Moreover, DECT boasts a lower radiation dose compared to traditional imaging methods. Studies have shown that the accuracy of identifying all PE locations exceeds 95% when using monoenergetic images at 40, 60, 70, and 100 keV [23].

As an advanced imaging modality, spectral detector computed tomography (SDCT) has gained increasing prominence in clinical practice. Scholars such as Mu and R have explored the utility of SDCT in distinguishing between lung adenocarcinoma and squamous cell carcinoma. Their study identified seven morphological CT features that significantly differed between AC and SC, including tumor location, lobulation, spinous processes, air bronchogram, vacuole sign, atelectasis and/or obstructive pneumonitis, and vascular invasion (all *p* values <0.05). Moreover, spectral parameters, including Z-eff, IC, normalized IC (NIC), and the spectral curve slope (λ) at 40-60 keV, were found to be higher in AC compared to SC (all *p* value <0.001) in both arterial and venous phases. The area under the ROC curve (AUC) for λ in the venous phase was 0.864, with a sensitivity of 85.8% and specificity of 74.3%. Combining morphological and spectral parameters increased the AUC to 0.946, with sensitivity and specificity reaching 89.8% and 87.1%, respectively. These findings highlight the significant diagnostic value of dual-energy spectral quantitative parameters in distinguishing between SC and AC, underscoring the potential of combining morphological and spectral parameters to enhance diagnostic accuracy [24]. Peña, J. A. and colleagues conducted a study comparing the efficacy of conventional CT parameters, such as HU and bone mineral density (BMD), with spectral CT parameters, including Z-eff and ECDM, in discerning between implant materials and bone tissue. Their findings revealed that Zeff exhibited a sharper distribution and greater discriminatory ability compared to ED, BMD, and HU. Particularly in the cancellous region of the femoral neck, Zeff demonstrated average AUC values of 0.996 and 0.998, respectively, indicating its exceptional performance in distinguishing AGN1 from bone tissue, approaching near-perfect accuracy. These results suggest the potential utility of this technology in patients undergoing similar implant treatments, thereby enhancing patient care and recovery processes [25].

Our study, albeit informative, is subject to several limitations as a single-center retrospective analysis: Firstly, the sample size is relatively modest, warranting further expansion for robust conclusions. Secondly, our findings necessitate validation across multiple centers to enhance generalizability. Thirdly, the potential for employing artificial intelligence and similar technologies for automated APE embolus identification warrants additional investigation. Notably, we observed conspicuous lesion areas indicative of APE on the Z-eff map, often exhibiting fan-shaped alterations, likely reflecting corresponding changes in the affected vasculature. This phenomenon merits further exploration.

## CONCLUSION

In Conclusion, SDCT emerges as a valuable adjunct for APE diagnosis. The Z-eff map offers intuitive visualization of lesion sites, while iodine density mapping, VMIs, and associated parameters facilitate quantitative analysis of these disparities. With the widespread application of SDCT, the diagnosis of APE has moved from a single CT value indicator in traditional CT to a multi-parameter era, which has a broad application prospect. The related technology is expected to enter clinical guidelines to serve patients and doctors.

## Figures and Tables

**Table 1:** Clinical and imaging characteristics in patients.

Variable Characteristic	Normal Area	APE related Lesions	P
Patients Age, year Sex Male Female	n=86		-
	28-87 (63±49)		
	-		
	53 (61.6%)		
	33 (38.4%)		
Normal field and APE lung field	Normal field n=85	Ape lung field n=85	-
40KeV	-652.732±165.344	-684.470±293.010	<0.001
70KeV	-754.697±116.277	-700.986±283.550	0.162
100KeV	-778.839±105.928	-693.446±294.888	0.902
Ratio	0.820±0.150	1.095±1.517	<0.001
SUB	126.107±71.202	8.974±112.144	<0.001
Z-effect	10.559±1.252	7.723±0.750	<0.001
IC	1.805±1.370	0.297±0.350	<0.001
ECDM	20.535±10.818	30.331±28.848	0.422
Normal blood vessels and embolism	Normal blood n=97	Pulmonary embolism n=97	-
40 KeV	1066.378±384.970	161.549±102.817	<0.001
70 KeV	336.907±151.389	57.055±42.887	<0.001
100 KeV	162.672±105.475	31.908±44.144	<0.001
Ratio	6.028±7.293	-12.072±158.857	<0.001
SUB	903.706±306.564	129.641±106.639	<0.001
Z-effect	11.599±0.899	8.224±0.710	<0.001
IC	12.326±3.940	1.746±1.420	<0.001
ECDM	105.455±3.806	101.561±5.045	<0.001

**Note: **All values are presented as mean±SD.

**Table 2:** ROC curve analysis of the identification of normal area and APE related lesions.

Parameters	Cut-off Value	AUC	Sensitivity (%)	Specificity (%)
Normal field and APE lung field	-	-	-	-
40KeV	-	0.695	-	-
Ratio	-	0.052	-	-
SUB	≥57.200	0.975	91.8	95.3
Z-effect	≥9.490	0.986	95.3	98.8
IC	≥0.655	0.974	94.1	91.8
Normal blood vessels and embolis	-	-	-	-
40KeV	≥517.000	0.990	99.0	100
70KeV	≥169.000	0.980	96.9	100
100KeV	≥82.800	0.962	95.9	99.0
Ratio	≥5.047	0.784	91.8	74.2
SUB	≥406.000	0.990	99.0	99.0
Z-effect	≥9.880	0.999	99.0	99.0
IC	≥5.470	1.000	100	99.0
ECDM	≥105.000	0.848	71.1	91.8

## Data Availability

All study data are included in the supplementary materials and are available upon request from the corresponding authors [B.H], [Y.L.W] and [F.L].

## LIST OF ABBREVIATIONS

CT: Computed Tomography
SDCT: Dual-layer Spectral CT
APE: Acute Pulmonary Embolism
VMI: Virtual Monoenergetic Image
IC: Iodine Concentration
ECDM: Electron Cloud Density Map
Z-eff: Effective Atomic Number
VMI slope: Hounsfield unit attenuation plots
SUB: Subtraction
ROC: Receiver Operating Characteristic Curves
CTA: CT Angiography
ESC: European Society of Cardiology
HU: Hounsfield Units
ROI: Regions of Interest
ICC: Interclass Correlation Coefficient
V/Q SPECT: Ventilation/Perfusion Single Photon Emission Computed Tomography
DECT: Dual-energy CT
PA: Pulmonary Artery

## STANDARDS OF REPORTING

STROBE guidelines were followed.
